# Micro-CT evaluation of apical enlargement of molar root canals using rotary or reciprocating heat-treated NiTi instruments

**DOI:** 10.1590/1678-7757-2018-0689

**Published:** 2019-07-25

**Authors:** Jáder Camilo PINTO, Mariana Mena Barreto PIVOTO-JOÃO, Camila Galetti ESPIR, Maria Luiza Gioster RAMOS, Juliane Maria GUERREIRO-TANOMARU, Mario TANOMARU-FILHO

**Affiliations:** 1 Universidade Estadual Paulista Faculdade de Odontologia de Araraquara Departamento de Odontologia Restauradora Araraquara São Paulo Brasil Universidade Estadual Paulista – UNESP, Faculdade de Odontologia de Araraquara, Departamento de Odontologia Restauradora, Araraquara, São Paulo, Brasil

**Keywords:** Dental pulp cavity, Root canal preparation, Dental instruments, X-ray computed tomography

## Abstract

**Objective:**

The aim of this study was to evaluate the root canal preparation and apical enlargement of molar root canals with rotary or reciprocating heat-treated nickel-titanium (NiTi) instruments, by using micro-computed tomography (micro-CT).

**Methodology:**

Mesial root canals (n=48) of mandibular molars, with a curvature between 20° and 40°, were prepared with ProDesign Logic (PDL) 25.01 and 25.06 in rotary motion, or ProDesign R (PDR) 25.06 in reciprocating motion (PDR). Apical enlargement was performed with PDL35.01 and PDL35.05 or PDR35.05. Scanning with 9 µm resolution was performed before and after preparation, and, after apical enlargement, by using micro-CT. The percentage of volume increase, debris and untouched root canal surface, transportation, centralization and preparation time were analyzed. ANOVA and Tukey or Kruskall-Wallis and Dunn statistical tests were conducted (α=.05).

**Results:**

PDL promoted a higher apical percentage of volume increase, and lower percentage of debris and untouched root canal surface than PDR 25.06 preparation in entire canal and in all thirds (P<.05). Apical enlargement with PDL 35.05 and PDR 35.05 produced a higher percentage of volume increase in the apical region in relation to the initial preparation (P<.05). PDR 35.05 and PDL 35.05 showed similar results in relation to percentage of debris and untouched root canal surface in entire canal and in all thirds (P>.05). Centralization and transportation showed no difference (P>.05). PDR required less time to perform preparation and apical enlargement (P<.05).

**Conclusions:**

The apical enlargement 35.05 with CM heat-treatment instruments using reciprocating and rotary motion reduced the percentage of debris and untouched root canal surface, without causing deviations or procedural errors. The protocol of greater apical enlargement favors the cleaning of the root canals in both kinematics. Preparation by the reciprocating system was faster than by the rotary system.

## Introduction

Endodontic treatment success depends on the cleaning and removal of microorganisms from the root canal system^1^ . The removal of infected dentin contributes to disinfection during root canal preparation^2^ . However, this preparation generally also results in a sizable percentage of non-instrumented surface^3 - 5^ .

Apical root canal enlargement favors cleaning and removal of infected dentin^6^ , decreased non-instrumented surface of root canals^5^ and enhanced performance of the irrigating solution^7^ . However, apical enlargement may also increase root canal transportation due to the reduced flexibility of instruments with larger diameter^8^ . Furthermore, excessive removal of dentin may decrease the strength of the tooth^9^ .

The kinematics and number of instruments used in instrumentation may influence the final quality of root canal preparation. Current literature did not show a significant difference in the Increase in canal volume between rotational and reciprocating preparation^10^ and transportation in mandibular molars have been found to be similar between rotary and reciprocating instruments^11^ . Nevertheless, some studies have reported a higher percentage of accumulated debris after instrumentation with reciprocating systems^12 , 13^ . Other studies have reported similar accumulation of debris for root canal preparation with both rotary and reciprocating files^6^ . The reciprocating motion has proven safer during root canal preparation^14^ . Furthermore, reciprocating systems can prepare root canals more quickly than rotary systems and with similar shaping ability^11 , 14^ .

The use of nickel-titanium (NiTi) instruments allows the apical enlargement of root canals, while maintaining their trajectory^9^ . Heat-treated NiTi instruments have better mechanical behavior, flexibility and cyclic fatigue resistance than conventional NiTi instruments, and can provide a centralized preparation in curved canals^3 , 16 - 19^ . However, CM heat treatment makes NiTi instruments more malleable, thus reducing hardness and possibly deforming the instrument^15^ . Although this thermal treatment causes NiTi instruments to lose hardness, previous studies have shown that both CM heat-treated NiTi instruments in rotary motion and other NiTI instruments leave the root canal surface similarly untouched^3 , 20^ . These instruments can maintain the trajectory of curved root canals^3 , 19^ . Current literature is scarce regarding the preparation with CM heat-treated NiTi instruments in reciprocating motion.

The Prodesign Logic (PDL, Easy Dental Equipment, Belo Horizonte, MG, Brazil) is a rotary system capable of preparing the root canal using two instruments: a glide path file, taper .01, and a shaping file, taper .05 or .06. PDL instruments showed higher cyclic fatigue resistance than WaveOne Gold (Dentsply Maillefer, Ballaigues, Switzerland)^21^ and are associated with the ability to maintain the root canal trajectory^19^ . A new reciprocating instrument manufactured from NiTi with CM heat treatment, the Prodesign R (PDR, Easy Dental Equipment, Belo Horizonte, MG, Brazil), was developed to be used in counter-clockwise reciprocating motion. The ideal reciprocating angles for the use of this instrument are not informed. These files are available in 25.06 and 35.05 diameters, and their cross section is “S” shaped. PDR reciprocating instruments have demonstrated low debris extrusion^16^ ; high flexural resistance in comparison to other heat-treated NiTi reciprocating instruments^15^ and little tendency to transport curved root canals^22^ .

The aim of this study was to evaluate the increase in volume, debris, untouched root canal surface, transportation, centralization and preparation time of root canals in the mesial roots of mandibular molars, after root canal preparation with the PDL rotary or the PDR reciprocating system, using file size 25.06 for preparation and up to size 35.05 for enlargement. The null hypothesis was that there is no difference between the root canal preparation systems in relation to the studied parameters.

## Methodology

The sample size for this study was calculated after estimating the effect size for the different variables (percentage of volumetric increase, debris and non-instrumented surface, centralization and transport). Tests were performed using specific software (G* Power 3.1.7 for Windows, Heinrich Heine, Universitat Düsseldorf, Düsseldorf, Germany). The effect size was specific for each variable, determined by previous studies^6 , 23 , 24^ that used micro-computed tomography (micro-CT) to evaluate root canals with similar morphology. Nineteen samples per group were indicated as the ideal required size. A sample of 24 root canals per group was stipulated, considering risk of tooth loss during the methodology.

After approval from the Ethics Committee of the Araraquara Dental School, Universidade Estadual de São Paulo (UNESP), Brazil (protocol number 1.968.137), mesiobuccal and mesiolingual root canals from mandibular molars were selected. A digital radiography system (Kodak RVG 6100, Kodak Dental Systems, NY, USA) and micro-CT were used to confirm the inclusion criteria and to perform a homogeneous distribution of the samples. The scanning was performed at 35 µm voxel size, using a computed microtomographic scan (Skyscan 1176, Bruker-MicroCT, Kontich, Belgium) with the following parameters: copper and aluminum filter, exposure time of 87 ms, frame 3, rotation of 360°, 80 kV, and 300 uA. A total of 24 first and second human mandibular molars with two mesial root canals, with type IV configuration according to the Vertucci classification^25^ , were selected. Complete apical formation, absence of root fractures, angle of curvature between 20° and 40°, in accordance with the Schneider method^26^ ,and radius of curvature smaller than 10 mm, following Pruett methodology^27^ , were observed. The tooth size was standardized at 18 mm, with a tolerance of ±2 mm of discrepancy. The selected root canals were stored in a 0.1% thymol solution at 5°C. The teeth were randomly divided into two experimental groups (n=24), with stratified random sampling, considering the volume of the preoperative root canals.

### Preoperative stage

After the specimens were washed in water for 48 hours, access to the canals was obtained with a high speed bur (n.2, KG Sorensen, São Paulo, Brazil), and the root canals were irrigated with 2.5% sodium hypochlorite. A size 10 K-file (Dentsply Sirona, Ballaigues, Switzerland) was used to explore the mesial root canals until its tip was visible through the apical foramen. The working length (WL) was established 1 mm short of the apical foramen. The specimens were instrumented with a size 10 K-file, using the balanced-force technique to perform the glide path, up to the apical foramen. The roots were embedded in condensation silicone (Oranwash, Zhermack SpA, Badia Polesine, Italy) to simulate the periodontal ligament. Afterwards, a single, experienced operator performed the root canal preparations.

### Operative stage

#### PDL Rotary Root Canal Preparation

The PDL instruments, size 25, .01 taper, were activated by an electric motor (VDW.SILVER, VDW GmbH, Munich, Germany) in rotary motion at 350 rpm and 1 N cm^- 1^ of torque in accordance with the manufacturer’s instructions, with in-and-out movements up to the WL. Then, a PDL instrument, size 25, .06 taper, was used at 600 rpm and 3 N cm^- 1^ of torque (manufacturer’s instructions for curved root canals), with movements in the apical direction up to the WL. A brushing motion was performed in the safety zone (mesial wall) with a mean amplitude of 3 mm, totaling three movements: mesial, mesiobuccal and mesiolingual. After concluding the operative procedure, the samples were scanned by using micro-CT with a 9 μm voxel size resolution.

The PDL instruments, size 35, .01 taper, were activated by an electric motor (VDW.SILVER, VDW GmbH, Munich, Germany) in rotary motion at 350 rpm and 1 N cm^- 1^ of torque, in accordance with the manufacturer’s instructions, with in-and-out movements up to the WL. Afterwards, a PDL instrument, size 35, .05 taper, was used at 600 rpm and 3 N cm^- 1^ of torque (manufacturer’s instructions for curved root canals), with movements in the apical direction up to the WL. A brushing motion was performed in the safety zone (mesial wall) with a mean amplitude of 3 mm, totaling three movements: mesial, mesiobuccal and mesiolingual. After concluding the operative procedure, the samples were scanned by micro-CT with a 9 μm voxel size resolution.

#### PDR Reciprocating Root Canal Preparation

The PDR instruments, size 25, .06 taper, were activated by an electric motor (VDW.SILVER, VDW GmbH, Munich, Germany) in “RECIPROC ALL” mode, in accordance with the manufacturer’s instructions. The instruments were inserted into the root canal by thirds (coronal, middle and apical) using an in-and-out movement, up to the WL. A brushing motion was performed in the safety zone (mesial wall) with a mean amplitude of 3 mm, totaling three movements: mesial, mesiovestibular and mesiobuccal. After concluding the operative procedure, the samples were scanned by micro-CT with a resolution of 9 μm of voxel size.

The PDR instruments, size 35, .05 taper, were activated by an electric motor (VDW.SILVER, VDW GmbH, Munich, Germany) in “RECIPROC ALL” mode, in accordance with the manufacturer’s instructions. The instruments were inserted into the root canal by thirds (coronal, middle and apical), using an in-and-out movement, up to the WL. A brushing motion was performed in the safety zone (mesial wall) with a mean amplitude of 3 mm, totaling three movements: mesial, mesiovestibular and mesiobuccal. After concluding the operative procedure, the samples were scanned by micro-CT with a 9 μm voxel size resolution.

At each stage of preparation, all instruments were cleaned with gauze moistened with distilled water. The root canals were irrigated with 3 ml of 2.5% sodium hypochlorite using a 30-G NaviTip needle (Ultradent Products, South Jordan, UT, USA), 2 mm short of the WL, after preparing each third with in-and-out movements. A total of 9 mL of NaOCl was used to irrigate each canal. Final irrigation was performed with 2.5 ml of 17% EDTA, under agitation for 3 minutes, with a gutta-percha cone size 25, and then, irrigation with 5 mL of 0.5% sodium hypochlorite. The preparation time was recorded using a chronometer, without considering the irrigation time in seconds.

## Micro-CT analysis

Analysis of the percentage of volume increase, debris and untouched root canal surface, transportation and centralization, was performed by scanning the samples by using a micro-CT (SkyScan 1176; Bruker Micro-CT, Kontich, Belgium) before and after preparation, and after apical enlargement, in which cases the samples were scanned in the same position in all the stages. The following parameters were used: copper and aluminum filter; 90 kV power; 278 mA energy; evolution cycle 180° and rotation 0.5, with 9 µm voxel size. The images obtained before and after preparation, and after apical enlargement, were reconstructed using NRecon software, and superimposed with geometric alignment using the Data Viewer software (Data Viewer v.1.5.1, Bruker, Kontich, Belgium). Quantitative analyses were then made using CTAn software (CTAn v.1.14.4, Bruker, Kontich, Belgium), by applying task lists with arithmetic and logic operations between the superimposed sections.

Each parameter was evaluated for the entire root canal and for each root canal third. A value of approximately 9 mm was determined for the total length analysis, and approximately 3 mm for each third. Aided by CTAn software, the bottom value corresponded to the WL, and 9 mm was added to this value to determine the top value.

Initial volume, final volume and final surface area after preparation were obtained. Based on these values, the percentage of volumetric increase (% Volumetric increase), percentage of debris (% Debris) and percentage of uninstrumented surface (% Uninstrumented surface) were calculated using the following formulas:^12^


$$\%\;\mathrm{Volumetric}\;\mathrm{increase}\;=\;\left[\frac{\mathrm{Final}\;\mathrm{volume}\;\times \;100}{\mathrm{Initial}\;\mathrm{volume}}\right]\;-\;100$$

$$\%\;\mathrm{Debris}\;=\;\frac{\mathrm{Volume}\;\mathrm{of}\;\mathrm{debris}\;\times \;100}{\mathrm{Final}\;\mathrm{volume}}$$

$$\%\;\mathrm{Uninstrumented}\;\mathrm{surface}\;=\;\frac{\mathrm{Uninstrumented}\;\mathrm{surface}\;\times \;100}{\mathrm{Final}\;\mathrm{surface}}$$

### Final surface

**Figure 1:**
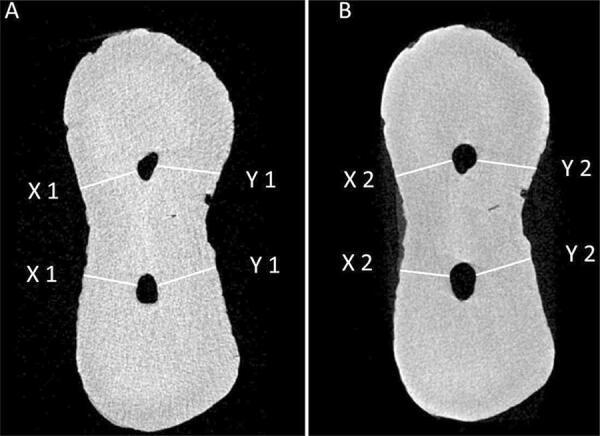
Representative micro-CT cross sections of the middle third of the PDL group, taken from the mesial root canals of mandibular molars, showing the shortest distance between the edge of the root and the canal, determined to perform the analyses of transportation and centralization in each group. A, preoperative root canal, and B, after preparation

Analyses of root canal transportation and centralization were made from the superimposed images, using the CTAn software, as previously described. The shortest distance between the mesial edge of the root and the canal before instrumentation (X1), to the shortest distance between the mesial edge of the root and the instrumented canal (X2), to the shortest distance between the distal edge of the root and the canal before instrumentation (Y1) and the shortest distance between the distal edge of the root and the instrumented canal (Y2) were measured, as proposed by Gambill, et al *.* ^28^ (1996) ( Figure 1 ). Five cross sections were measured for each third (coronal, middle and apical), and were determined by the arithmetical mean value. The thirds corresponded to 3, 6 and 9 mm from the anatomic apex, with each third covering 3 mm of the extension of the canal. The degree of canal centralization was obtained by means of the following equation: (X1-X2)/(Y1-Y2), and the root canal transportation was obtained by means of the following equation: (X1-X2) – (Y1-Y2)^28^ . Centralization data were ordered from 0 to 1, with values closest to 1 being completely centralized, and those closest to zero being completely outside centralization. In scoring the data for the deviation, the closer the values to zero, the smaller the deviation. Negative numbers represented deviation in the mesial direction, and positive numbers, in the distal direction.

### Statistical analysis

The data obtained for each of the parameters evaluated were submitted to the Shapiro-Wilks normality test. The data obtained on the evaluation of increased volume, debris, untouched root canal surface and transportation showed a non-normal distribution, and the data obtained on the canal volume, centralization and preparation time showed normal distribution. Thus, the Kruskal-Wallis and Dunn tests were used to compare the groups concerning increased volume, debris, untouched root canal surface and transportation values. Considering the data for canal volume, centralization and preparation time, the parametric ANOVA and Tukey tests were used. The significance level adopted was 5% for both analyses.

## Results

**Figure 2:**
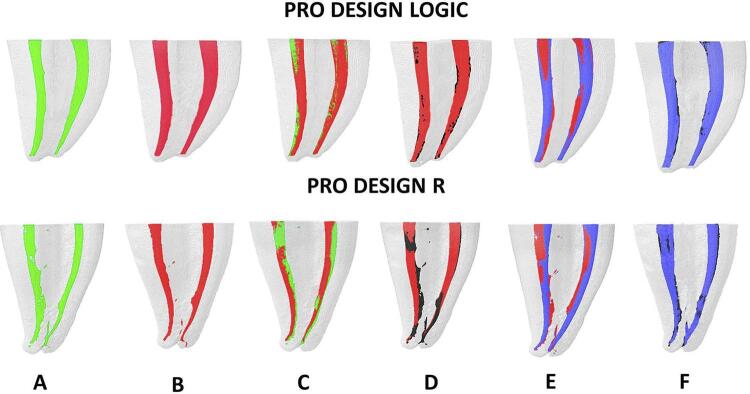
Micro-CT rendering of mandibular molar mesial canals prepared by using PDL and PDR, showing: A, preoperative canal (green); B, post-instrumentation (red); C, superimposition of preparation on initial canal; D, debris (black); E, superimposition of the apical enlargement (purple) on preparation; and F, apical enlargement with debris

Table 1 shows no difference in percentage of volumetric increase between the PDL 25.06 and PDR 25.06 instruments in relation to total volume (P>.05); however, PDL 25.06 showed a percentage of volumetric increase higher than PDR 25.06 in the apical third (P<.05) ( Figure 2 ). PDL 25.06 showed a lower percentage of debris and untouched root canal surface than PDR 25.06 in the entire canal and in all thirds (P<.05).

**Table 1:** Root canal volume, increase in volume (%), debris (%), untouched root canal surface (%), centralization, transport and preparation time after preparation with ProDesign Logic 25.06 and Processing R 25.06 instruments

		Preparation in relation to pre-operative canal	
		ProDesign Logic 25.06	ProDesign R 25.06
Pre-operative canal (mm^3^)	Total*	1.75±0.72^a^	1.62±0.41^a^
	Coronal*	0.97±0.43^aA^	0.87±0.23^aA^
	Middle*	0.58±0.26^aB^	0.52±0.18^aB^
	Apical*	0.27±0.11^aC^	0.23±0.06^aC^
Preparation 25.06 (mm^3^)	Total*	2.94±0.65^a^	2.51±0.27^a^
	Coronal*	1.71±0.38^aA^	1.45±0.18^aA^
	Midlle*	0.94±0.20^aB^	0.80±0.07^aB^
	Apical*	0.45±0.09aC	0.30±0.07^bC^
Increase in Volume (%)	Total**	65.88 (20.33-329.6)^a^	56.65 (14.34-167.1)^a^
	Cervical**	75.25 (22.52-388.9)^aA^	66.92 (8.361-203.3)^aA^
	Médio**	63.43 (8.463-724.0)^aA^	54.76 (7.754-196.4)^aAC^
	Apical**	66.15 (2.165-339.0)^aA^	33.15 (0.5198-143.3)^bBC^
Debris (%)	Total**	3.974 (0.08753-13.03)^b^	8.760 (2,702-17.29)^a^
	Cervical**	3.470 (0.06563-12.10)^bA^	7.116 (3.117 -14.90)^aAC^
	Médio**	2.238 (0.05340-17.47)^bA^	6.278 (0.1008-15.10)^aBC^
	Apical**	4.205 (0.08676-15.66)^bA^	12.21 (1.533-17.87)^aA^
Untouched surface (%)	Total**	22.51 (1.262-62.62)^b^	50.36 (17.70-76.12)^a^
	Cervical**	23.46 (1.488-49.11)^bA^	36.04 (4.287-60.57)^aA^
	Médio**	22.82 (0.8117-47.83)^bA^	47.77 (5.685-60.66)^aA^
	Apical**	21.34 (2.019-54.19)^bA^	48.53 (17.58-67.65)^aA^
Centralization	Cervical*	0.5836 ±0.2643^aA^	0.6132±2773^aA^
	Médio*	0.5362 ±0.2546^aA^	0.6270±0.2188^aA^
	Apical*	0.5688 ±0.2273^aA^	0.5880±0.1810^aA^
Transport (mm)	Cervical**	0.0572 (-0.3126-0.5854)^aA^	0.03137 (-0.1440-0.3046)^aA^
	Médio**	0.0190 (-0.1194-3.3124)^aA^	-0.0170 (-0.0908-0.2360)^aBC^
	Apical**	0.00535 (-0.07726-0.1738)^aA^	0.02405 (-0.0726 -0.1738)^aAC^
Preparation Time (s)	Total*	85.47±25.81^a^	35.50±9.523^b^

Different superscript lowercase letters in the same line indicate statistical difference between the groups.

Superscript uppercase letters in the same column indicate statistical difference among the thirds of the same preparation for each analysis: mean and ± standard deviation for the parametric data (ANOVA* and Tukey Tests, 5% significance), and median, maximum and minimum values for the non-parametric data (Kruskal-Wallis** and Dunn, 5% significance)

Table 2 shows the comparison of the apical enlargement (35.05) related to the preparation (25.06). This analysis indicates no difference between the tested instruments in relation to percentage of volumetric increase, debris and untouched root canal surface in the entire canal and in all thirds (P>.05). Both PDL and PDR showed a higher percentage of volumetric increase in the apical third than the other thirds (P<.05).

**Table 2:** – Root canal volume, increase in volume (%), debris (%), untouched root canal surface (%), centralization, transport and preparation time, in the apical enlargement (35.05) in relation to preparation (25.06)

		Apical enlargement in relation to preparation	
		ProDesign Logic 35.05	ProDesign R 35.05
Preparation 25.06 (mm^3^)	Total*	2.94±0.65^a^	2.51±0.27^a^
	Coronal*	1.71±0.38^aA^	1.45±0.18^aA^
	Middle*	0.94±0.20^aB^	0.80±0.07^aB^
	Apical*	0.45±0.09^aC^	0.30±0.07^bC^
Apical enlargement 35.05 (mm^3^)	Total*	3.75±0.5^2a^	3.14±0.53^a^
	Coronal*	2.08±0.06^aA^	1.76±0.37^aA^
	Midlle*	1.12±0.19^aB^	0.99±0.26^aB^
	Apical*	0.69±0.10^aC^	0.39±.0.09^bC^
Increase in Volume (%)	Total**	20.40 (6,20-34.63)^a^	18.73 (9.75-35.46)^a^
	Cervical**	11.37 (2.31-45.05)^aB^	11.92 (2.74-35.67)^aB^
	Médio**	13.87 (0.26-47.37)^aB^	17.43 (4.27-38.44)^aB^
	Apical**	36.87 (11.75-92.34)^aA^	39.30 (10.66-88.32)^aA^
Debris (%)	Total**	3.30 (1,00-9.33)^a^	3.36 (0.12-27.89)^a^
	Cervical**	5.74 (0.048-11.70)^aA^	3.76 (0.48-21.82)^aA^
	Médio**	2.80 (0.08-8.98)^aA^	1.54 (0.10-21.33)^aA^
	Apical**	3.00 (0.17-18.35)^aA^	3.98 (0.29-19.32)^aA^
Untouched surface (%)	Total**	34.16 (10.75-57.53)^a^	31.17 (5.42-59.91)^a^
	Cervical**	42.37 (3.74-66.88)^aA^	30.17 (4.46-66.11)^aA^
	Médio**	30.33 (6.36-67.24)^aA^	20.39 (2.62-53.44)^aA^
	Apical**	25.39 (4.83-62.75)^aA^	21.87 (0.83-63.46)^aA^
Centralization	Cervical*	0.6100 ±0.2419^aA^	0.5733 ±0.2197^aA^
	Médio*	0.5905 ±0.6792^aA^	0.5130 ±6940^aA^
	Apical*	0.5801 ±0.2364^aA^	0.5998 ±0.2497^aA^
Transport (mm)	Cervical**	0.0384 (-0.05-0.2108)^aA^	0.0142 (-0.134-3448)^aA^
	Médio**	-0.0020 (-0.7886 -0.0882)^aAC^	0.0064 (-0.0376-0.1766)^aA^
	Apical**	-0.0114 (-0.6068-0.2478)^aBC^	0.0156 (-0.9596-0.2466)^aA^
Preparation Time (s)	Total*	87.37±19.64^a^	32.44±4.927^b^

Different superscript lowercase letters in the same line indicate statistical difference between the groups.

Table 3 shows the comparison of apical enlargement (35.05) with the preoperative canal. In this analysis, PDL achieved greater enlargement in the middle and apical thirds, compared with PDR (P<.05). There was no difference between the groups concerning debris and untouched root canal surface in the entire canal and in all thirds (P>.05).

**Table 3:** – Root canal volume, increase in volume (%), debris (%), untouched root canal surface (%), centralization, transport and preparation time, in the apical enlargement (35.05) in relation to pre-operative canal

		Apical enlargement in relation to pre-operative canal	
		ProDesign Logic 35.05	ProDesign R 35.05
Pre-operative canal	Total*	1.75±0.72^a^	1.62±0.41^a^
	Coronal*	0.97±0.43^aA^	0.87±0.23^aA^
	Middle*	0.58±0.26^aB^	0.52±0.18^aB^
	Apical*	0.27±0.11^aC^	0.23±0.06^aC^
Apical enlargement 35.05 (mm^3^)	Total*	3.75±0.52^a^	3.14±0.53^a^
	Coronal*	2.08±0.06^aA^	1.76±0.37^aA^
	Midlle*	1.12±0.19^aB^	0.99±0.26^aB^
	Apical*	0.69±0.10^aC^	0.39±.0.09^bC^
Increase in Volume (%)	Total**	107.50 (39.11-398.3)^a^	93.49 (37.99-184.40)^a^
	Cervical**	115.80 (37.49-238.00)^aA^	106.40 (23.36-256.00)^aA^
	Médio**	105.90 (26.01-477.50)^aA^	78.30 (16.31-210.90)^bA^
	Apical**	183.30 (27.74-433.20)^aA^	84.00 (8.44-176.20)^bA^
Debris (%)	Total**	0.97 (0.14-0.91)^a^	1.84 (0.17-17.76)^a^
	Cervical**	1.01 (0.03-9.98)^aB^	1.72 (0.05-9.33)^aA^
	Médio**	0.92 (0.02-9.96)^aB^	1.68 (0.19-9.96)^aA^
	Apical**	2.76 (0.01-12.90)^aA^	2.01 (0.07-14.90)^aA^
Untouched surface (%)	Total**	24.49 (2.56-53.57)^a^	27.23 (7.02-49.64)^a^
	Cervical**	21.24 (4.14-47.42)^aA^	24.56 (4.61-56.97)^aA^
	Médio**	21.97 (2.39-45.28)^aA^	26.53 (0.48-53.11)^aA^
	Apical**	20.03 (0.59-55.86)^bA^	31.53 (4.34-54.02)^aA^
Centralization	Cervical*	0.5444±0.2405^aA^	0.5885±0.2398^aA^
	Médio*	0.5785±0.2163^aA^	0.6018 ±0.1861^aA^
	Apical*	0.5990±0.2551^aA^	0.5873±0.2690^aA^
Transport (mm)	Cervical**	0.01736 (-0.993-0.1820)^aA^	0.01345 (-0.8278-0.1958)^aA^
	Médio**	0.05815 (-0.7358-0.8768)^aA^	-0.0193 (-0.1268-0.8810)^aA^
	Apical**	-0.0143 (-0.9596-0.2466)^aB^	0.0020 (-0.7064-0.9040)^aA^
Preparation Time (s)	Total*	173.00±41.22^a^	67.93±12.02^b^

Different superscript lowercase letters in the same line indicate statistical difference between the groups.

PDL promoted greater volume in the apical third than PDR in preparation (25.06) and in apical enlargement (35.05) (P<.05). There was no difference in centralization and transportation between the PDL and PDR instruments, after performing both preparations for 25.06 ( Table 1 , Figure 2 ), and after apical enlargement to 35.05. (P>.05) ( Table 2 , Figure 2 ). The preparation time with PDR was lower for preparing with instruments 25.06 ( Table 1 ), and also for apical enlargement with instruments 35.05 (P<.05) ( Tables 2 and 3 ).

## Discussion

This study assessed curved mesial roots of mandibular molars, regarding root canal preparation and apical enlargement using different instruments. The difficulty encountered in standardizing these teeth may have influenced the results^19^ . Despite these limitations, the use of extracted teeth seems to be the best option, since resin-made teeth have a critical limitation regarding the difference in hardness between dentin and resin^29^ . In addition, micro-CT was used in this study to select the teeth according to morphology, degree of curvature and volume of the root canals, thus determining the correct distribution between the groups. This selection, using a 35µm resolution scan, allows the analysis of pre-operative morphological parameters. Homogenization of the samples can be observed in Table 1 , with no difference in relation to the volume of the preoperative root canals. However, although the curvature and size of the roots were considered in the sample selection, only the volume was used to distribute the samples, thus constituting one of the main limitations of the study.

Residual infection after root canal preparation may prevent periapical healing^1^ . Enlargement of apical preparation can improve root canal disinfection^30^ . In this study, CM heat-treated NiTi instruments used in rotary kinematics promoted greater volume in the apical third in preparations with 25.06 instruments and in apical enlargement with 35.05 instruments, compared with CM heat-treated NiTi instruments used in reciprocating motion. Literature has shown that there is no difference in the volume increase for root canals prepared by rotary or reciprocating files^10^ . However, most studies compared instruments with different tapers^5 , 9 , 20 , 22^ and heat-treatments^11 , 18 , 20 , 22 , 33^ . In this study, the instruments were evaluated with the same size and similar NiTi heat-treatment. A previous study^22^ showed that the root canals prepared by PDR 25.06 showed lower final volume than Reciproc 25.08 (VDW, GmbH, Munich, Germany) and Mtwo 25.06 (VDW, GmbH, Munich, Germany).

The use of more instruments using the rotary protocol preparation may have favored the greater enlargement of the apical third. The glide path instrument may reduce the stress in shaping instruments by increasing the preparation capacity for the apical third. Considering that apical NiTi instruments undergo greater mechanical stress^31 , 32^ , a glide path could help decrease stress in the shaping instrument^21 , 31 , 32^ . The higher flexibility of CM heat-treated instruments makes the tensions generated in root canal preparation even more critical. Camargo, et al.^22^ (2018) reported that the Pathfile size 13.02 rotary instrument had to be used before using PRD 25.06 when preparing second mesiobuccal canals of maxillary first molars. The authors attribute the importance of the glide path instrument to the anatomical challenges of the root canal and the greater flexibility of CM heat-treated NiTi instruments, in relation to conventional NiTi instruments. The PDR instrument showed larger deformation capacity than WaveOne Gold (Dentsply/Tulsa Dental Specialties, Tulsa, OK, USA) and Reciproc Blue (VDW, Munich, Germany), both of which are instruments with lower torsional load^15^ . Therefore, an initial pathway in the root canal could reduce torsional stress^15^ . It should be borne in mind that the preparation of root canals may be influenced by the kinematics (rotary or reciprocating)^23 , 33^ and the number of instruments used^33^ .

The presence of debris in the root canal makes it difficult not only to disinfect the canal, but also to promote filling sealer adhesion to the dentinal tubules^24 , 34^ . After preparation to a size 25.06, a lower percentage of debris was observed for the PDL 25.06 than for the PDR 25.06, especially in the apical third, in which debris rates of 12.21% were observed for PDR and 4.20% for PDL. The use of rotary systems with a sequence of instruments promoted a larger amount of debris removal^12 , 13 , 35 , 36^ . The continuous movement of rotary instruments favors the removal of debris through the space between the instruments and the canal, whereas reciprocating motion may lead to retention of debris^12 , 35^ . In addition, the higher percentage of volumetric increase promoted by rotary instruments may have contributed to the lower accumulation of debris. After apical enlargement, the systems show a small and similar amount of debris. Enlargement of the apical root canal improves the physical effect of the irrigant solution and removal of debris^7^ . A study using micro-CT analysis showed a smaller percentage of debris after apical enlargement^6^ .

A higher percentage of untouched root canal surface was observed for the PDR 25.06 reciprocating system, with a total median of 50.36%, representing twice the percentage value of untouched area for the PDL system (22.57%). This result may be related to use of the PDL 25.01 glide path instrument in the rotary protocol. The smaller diameter of the instrument allowed the cleaning of irregularities and areas of difficult access^13^ . However, after apical enlargement from 25.06 to 35.05, the percentage of surface untouched by the PDR instrument decreased to median values of 31.17% for the apical enlargement in relation to the preparation, and 27.3% for the apical enlargement in relation to the preoperative root canal. Significant reduction in untouched root canal surface was observed with the enlargement of apical preparation^5^ . These results confirm that an apical enlargement promotes smaller untouched dentinal wall surface, even in the mesial canals of mandibular molars, and better apical cleaning, as previously observed^37^ . Even though apical enlargement with PDL 35.05 produced greater volume in the apical third, there were no differences between the groups concerning the untouched root canal surface. Previous studies found no correlation between volumetric increase and non-instrumented surface of root canals^20 , 33^ . It should be borne in mind that the untouched root canal surface may be critical for root canal disinfection, since infected dentin may retain bacteria^38^ . It may be assumed that the lower volume obtained by apical enlargement (35.05) using the PDR instruments did not affect the final quality of the preparation in relation to the PDL instruments. Studies that used micro-CT have demonstrated a high percentage of untouched walls due to anatomical complexities, irrespective of the system used^3 , 4^ .

Transportation and centralization may be quantified by means of superimposed images of the canal^18 , 19^ . In this study, both preparations were able to maintain the canal centralized, without deviations, even in apical enlargement. The mean transportation observed in this study ranged from -0.002 to 0.0581 mm, which is below the acceptable limit of 0.15 mm^39^ . Heat-treated NiTi instruments are more flexible, and are therefore more capable of maintaining the canal more centralized^19^ , with less transportation of the root canal^3 , 15 , 17 - 19^ . Pinheiro, et al.^19^ (2018) evaluated apical transportation and centralization in the mesial roots of mandibular molars by micro-CT, and found that CM heat-treated instruments, PDL, ProDesign S (Easy Dental Equipment, Belo Horizonte, MG, Brazil), HyFlex CM (Coltene-Whaledent, Allstetten, Switzerland) and HyFlex EDM (Coltene-Whaledent, Altstatten, Switzerland) promoted little apical transportation and were able to maintain a curved root canal trajectory.

Canal preparation with PDR files was performed more quickly than with PDL, in both initial instrumentation and additional enlargement, corroborating the findings of different studies comparing rotary and reciprocating preparation^12 , 14^ . The PDR reciprocating system is composed of only one instrument, whereas the PDL system has two instruments, one used as a glide path and the other, for shaping. In disagreement with our study, Menezes, et al.^21^ (2017) showed a shorter instrumentation time for PDL compared with PDR. However, acrylic resin artificial canals were used, whereas extracted human mandibular molars were used in our study.

The root canal preparation is critical to the success of endodontic treatment^3^ . The preparation of curved canals with greater apical enlargement presents a challenge^9^ . However, the results of this study demonstrate that heat-treated instruments promoted centralized preparation using reciprocating or rotary motions, even after the apical increase (35.05). These protocols promoted reduction of debris and surface of the untouched root canal, which may be related to better root canal disinfection^30^ . The apical enlargement to size 35.05 with CM heat-treated instruments using reciprocating and rotary motion seems a viable clinical procedure for the prognosis of the endodontic treatment.

## Conclusions

The apical enlargement 35.05 with CM heat-treatment instruments using reciprocating and rotary motion reduced the percentage of debris and untouched root canal surface, without causing deviations or procedural errors. The protocol of greater apical enlargement favors the cleaning of the root canals in both kinematics. Preparation by the reciprocating system was faster than by the rotary system.
